# Differential profile of protein expression on human keratocytes treated with autologous serum and plasma rich in growth factors (PRGF)

**DOI:** 10.1371/journal.pone.0205073

**Published:** 2018-10-12

**Authors:** Eduardo Anitua, María de la Fuente, Francisco Muruzabal, Ronald Mauricio Sánchez-Ávila, Jesús Merayo-Lloves, Mikel Azkargorta, Felix Elortza, Gorka Orive

**Affiliations:** 1 BTI—Biotechnology Institute, Vitoria-Gasteiz, Spain; 2 University Institute for Regenerative Medicine and Oral Implantology—UIRMI (UPV/EHU-Fundación Eduardo Anitua), Vitoria-Gasteiz, Spain; 3 Instituto Universitario Fernández-Vega, Universidad de Oviedo, Oviedo, Spain; 4 Proteomics Platform, CIC bioGUNE, CIBERehd, ProteoRed-ISCIII, Bizkaia Science and Technology Park, Derio, Spain; 5 NanoBioCel Group, Laboratory of Pharmaceutics, School of Pharmacy, University of the Basque Country UPV/EHU, Paseo de la Universidad 7, Vitoria-Gasteiz, Spain; 6 Biomedical Research Networking Centre in Bioengineering, Biomaterials, and Nanomedicine (CIBER-BBN), Vitoria-Gasteiz, Spain; University of Florida, UNITED STATES

## Abstract

**Purpose:**

The main objective of this study is to compare the protein expression of human keratocytes treated with Plasma rich in growth factors (PRGF) or autologous serum (AS) and previously induced to myofibroblast by TGF-β1 treatment.

**Methods:**

Blood from healthy donor was collected and processed to obtain AS and PRGF eye drops. Blood derivates were aliquoted and stored at -80°C until use. Keratocyte cells were pretreated for 60 minutes with 2.5 ng/ml TGF-β1. After that, cells were treated with PRGF, AS or with TGF-β1 (control). To characterize the proteins deregulated after PRGF and AS treatment, a proteomic approach that combines 1D-SDS–PAGE approach followed by LC–MS/MS was carried out.

**Results:**

Results show a catalogue of key proteins in close contact with a myofibroblastic differentiated phenotype in AS treated-cells, whereas PRGF-treated cells show attenuation on this phenotype. The number of proteins downregulated after PRGF treatment or upregulated in AS-treated cells suggest a close relationship between AS-treated cells and cytoskeletal functions. On the other hand, proteins upregulated after PRGF-treatment or downregulated in AS-treated cells reveal a greater association with processes such as protein synthesis, proliferation and cellular motility.

**Conclusion:**

This proteomic analysis helps to understand the molecular events underlying AS and PRGF-driven tissue regeneration processes, providing new evidence that comes along with the modulation of TGF-β1 activity and the reversion of the myofibroblastic phenotype by PRGF.

## Introduction

The increase of population age is associated with an augmentation of the ophthalmological problems concerning the ocular surface and the cornea. These complications are mainly due to age-specific hormonal changes, autoimmune diseases (as rheumatoid arthritis or systemic lupus erythematosus) or are also caused by local infections [1–3].

The treatment of ocular surface disorders (OSDs) could be addressed in a step by step approach implying topic and local medical therapy as lubricating artificial tears, anti-inflammatory substances (corticoids or cyclosporine or secretagogues) and, blood derived eye drops such as autologous serum and platelet rich plasmas (PRP), including Plasma Rich in Growth Factors (PRGF) eye drops [4–8].

New interesting regenerative therapies for the treatment of different pathologies are used today in the ophthalmology field including amniotic membrane transplantation (AMT) and the use of recombinant growth factors [9]. However, the uncertain results obtained after the application of AMT, the elevated manufacturing costs and the scarce clinical results, make it necessary to explore other therapeutic alternatives for corneal regeneration [10–12]. In this sense, PRGF-Endoret arises as a very promising therapeutic option with proven efficacy both *in vitro* and *in vivo* [13].

Corneal surface regenerative process depends on a myriad of interacting growth factors (epidermal growth factor (EGF), transforming growth factor beta (TGF-β1), keratinocyte growth factor (KGF), hepatocyte growth factor (HGF), platelet derived growth factor (PDGF) and fibroblast growth factor (FGF) among others) that coordinate biological events leading to an appropriate tissue regeneration [14]. Autologous serum (AS) has been classically employed for ocular surface disorders (OSD) due to its content in growth factors. The lack of a standardized manufacturing protocol, the high content in pro-inflammatory molecules (metalloproteinases and hydrolases) together with microbial contamination elevated risk, provide controversial AS clinical results [15, 16]. In order to avoid these limitations, a protocolized and standardized technology has been developed, PRGF eye drops, an autologous hemoderivate obtained by means of a closed system that includes platelet activation, avoiding leukocytes and pro-inflammatory molecules and with higher growth factor content than autologous serum. Other important features of PRGF eye drops in tissue regeneration relies on its bacteriostatic/bactericidal activity, its anti-fibrotic and anti-inflammatory potential, and its proven biological stability for at least 6 months [17]. *In vitro* studies have highlighted the beneficial effects of PRGF eye drops on human primary ocular surface cells accelerating corneal wound closure and increasing corneal epithelial cell proliferation and migration [18, 19]. An *in vivo* study demonstrated that PRGF eye drops reduced the re-epithelization time in comparison to a platelet rich plasma without platelet activation and compared to autologous serum [20]. In the case of a stromal injury, some fibroblasts develop actin contractile filaments and differentiate into myofibroblasts. However, the persistence of myofibroblastic cells after wound healing lead to the development of a corneal fibrotic scar and haze. PRGF eye drops exerts a protective effect against fibrotic scars formation by avoiding the transformation of TGF-β1-treated stromal fibroblasts to myofibroblasts [21], suggesting the beneficial effects of PRGF to promote corneal regeneration and minimizing scar formation [20, 22, 23].

PRGF eye drops low cost and easy production and its demonstrated ophthalmic clinical efficacy converts this autologous technology in a new promising human therapy over other hemoderivate products like autologous serum [24–28]. In this way, proteomic characterization of PRGF-treated cells could help to understand regenerative therapy processes and mechanisms and contribute in the optimization of eye drops application and dosage.

In the present study, the differential protein expression of TGF-β1-induced myofibroblasts treated with either Plasma rich in growth factors (PRGF) or autologous serum (AS) was determined. The latter could shed light on corneal haze reduction after PRGF application.

## Materials and methods

In order to use human-based PRGF, this study was approved by the Ethics Committee of the Eduardo Anitua Foundation for biomedical research on March 17, 2015. The ethics approval was obtained prior to the start of the experimental study. With the aim of characterizing the proteomic profile of human keratocytes after treatment with PRGF eye drops and autologous serum (AS), they were stimulated with TGF-β1 for differentiating them to myofibroblasts and then were treated with PRGF or AS to reverse the myofibroblastic phenotype. Corneal stromal keratocytes cells (termed HK) were pretreated with TGF-β1 + 0.1% FBS and afterwards were divided in 3 treatment groups: PRGF-Endoret + TGF-β1 or AS + TGF-β1, using TGF-β1 + 0.1% FBS as a control. Then, cell sediments were obtained and samples were subjected to the FAST protocol for protein digestion, being the obtained proteins identified by LC-MS (Liquid Chromatography–Mass Spectrometry) [29] and further quantified in order to dig into protein expression differences triggered by PRGF and AS treatments. Protein functional analyses were carried out for a deeper understanding of the molecular events underlying such changes.

### PRGF and autologous serum (AS) preparations

Blood from one healthy young male donor was harvested after informed consent into 9-mL tubes with 3.8% (wt/v) sodium citrate or in serum collection tubes (Z Serum Clot activator, Vacuette, GmbH, Kremsmünster, Austria). The study was performed following the principles of the Declaration of Helsinki. Blood sample for PRGF was centrifuged at 580 g for 8 min at room temperature in an Endoret System centrifuge (BTI Biotechnology Institute, S.L., Miñano, Álava, Spain); the whole plasma column over the buffy coat was collected using Endoret ophthalmology kit (BTI Biotechnology Institute, S.L., Miñano, Álava, Spain) avoiding leukocytes collection. The obtained PRGF supernatants were filtered, aliquoted and stored at -80°C until use. Blood sample for autologous serum preparation was allowed to clot at room temperature for 20 minutes and subsequently centrifuged for 10 min at 2000 g; after that, serum was collected and filtered by 0.22 μm PVDF filters. Then, total serum was diluted to 20% with sterile serum saline, aliquoted and stored at -80°C until use (termed AS).

### Cells

Cells involved in assays were corneal stromal keratocytes (termed HK) (ScienCell Research Laboratories, San Diego, CA) that were cultured according to manufacturer’s instructions. Briefly, cells were maintained in culture until confluence in Fibroblast medium supplemented with Fibroblast Growth Supplement (FGS), 2% fetal bovine serum (FBS) and antibiotics (penicillin/streptomycin) (ScienCell Research Laboratories, San Diego, CA, USA) and then were detached with animal origin-free trypsin-like enzyme (TrypLE Select, Gibco-Invitrogen, Grand Island, NY, USA). Cell viability was assessed by trypan blue dye exclusion. Passage 4 cells were used in all experiments.

### Myofibroblast differentiation: Reversion assay

In order to test the capacity of reversion of PRGF versus AS, HK cells were pretreated for 60 minutes with 2.5 ng/ml TGF-β1 + 0.1% FBS (TGF-β1, Chemicon-Millipore, Billerica, MA, USA); afterwards, medium was removed and wells were washed with phosphate buffered saline (PBS) in order to place the treatments for 30 minutes: 20% PRGF + 2.5 ng/ml TGF-β1 (termed PRGF) or 20% AS + 2.5 ng/ml TGF-β1 (termed AS), 2.5 ng/ml TGF-β1 + 0.1% FBS (termed control) was used as a control. Then, culture media were discarded and wells were rinsed with PBS and cells were collected with TrypLE. After centrifugation at 1500 rpm for 5 min, the cell pellets were incubated for 1h at room temperature with cell lysis buffer consisting on Tris 30 mM, thiourea 2 M, urea 7 M, CHAPS 4% (all from Sigma–Aldrich, St Louis, MO, USA) and distilled water in order to obtain the proteins. Finally, the supernatants were collected after centrifugation at 13000 rpm for 5 min, aliquoted and stored at -20°C until use. Samples were assayed in quadruplicate for each treatment.

**Fig 1:**
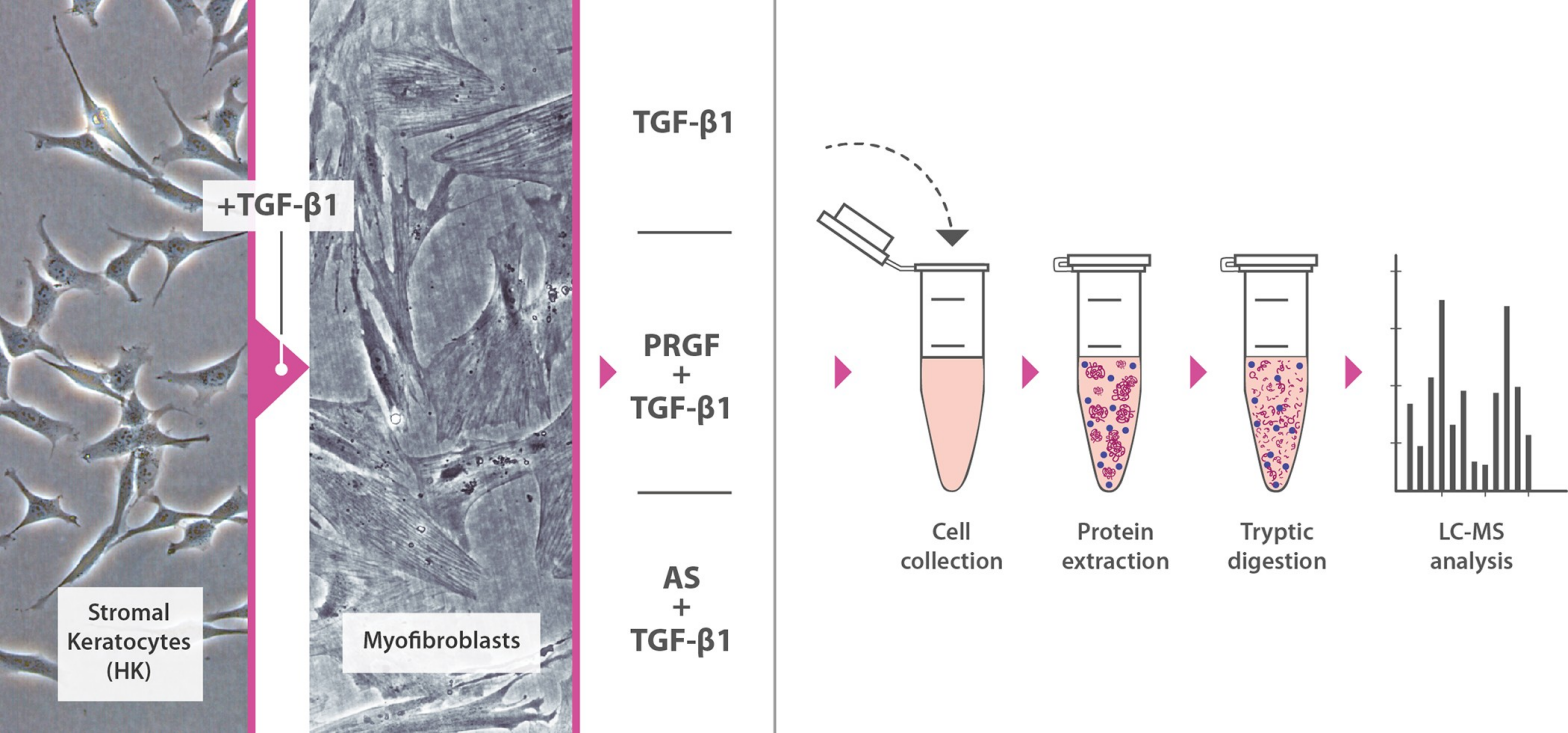
Human keratocytes were stimulated with TGF-β1 in order to induce a myofibroblastic phenotype and then were treated with TGF-β1 alone or in combination with PRGF or AS eye drops. After cell collection and protein extraction, tryptic digestion and Liquid chromatography–mass spectrometry (LC-MS) analysis were performed.

Fig 1 summarizes the general schema of the followed steps. Stromal keratocytes were treated with TGF-β1 in order to transform them to myofibroblasts and then reversion assay was performed; PRGF and AS treatments were analyzed to find differences in protein expression with mass spectrometry (LC-MS analysis).

### Protein digestion and LC-MS analysis (Liquid chromatography–mass spectrometry)

Samples were digested following the FASP protocol [29] with minor variations. Peptides were cleaned up with C18 Zip Tip stage tips (Chemicon-Millipore, Billerica, MA, USA) and loaded onto a nanoACQUITY UPLC System (Waters Corporation, Milford, MA, USA) connected to an LTQ Orbitrap XL mass spectrometer (Thermo Fisher Scientific, Waltham, MA, USA). An aliquot of each sample was loaded onto a Symmetry 300 C18 UPLC Trap column (180 μm x 20 mm, 5 μm (Waters Corporation, Milford, MA, USA)). The precolumn was connected to a BEH130 C18 column, 75 μm x 200 mm, 1.7 μm (Waters Corporation, Milford, MA, USA), and equilibrated in 3% acetonitrile and 0.1% aqueous formic acid. Peptides were eluted directly into the nanoelectrospray capillary (Proxeon Biosystems, Thermo Fisher Scientific, Waltham, MA, USA) at 300 nl/min, using a 30 min linear gradient of 3–50% acetonitrile for DIGE spots, or a 60 min linear gradient of 3–50% acetonitrile for LF samples. The mass spectrometer automatically switched between MS and MS/MS acquisition in DDA mode. Full MS scan survey spectra (m/z 400–2000) were acquired in the orbitrap with mass resolution of 30000 at m/z 400. After each survey scan, the six most intense ions above 1000 counts were sequentially subjected to collision-induced dissociation (CID) in the linear ion trap. Precursors with charge states of 2 and 3 were specifically selected for CID. Peptides were excluded from further analysis during 60 s using the dynamic exclusion feature.

### Differential analysis

Progenesis LC-MS (version 4.0.4265.42984, Nonlinear Dynamics) was used for the label-free differential protein expression analysis. After importing the Raw files from the MS acquisition of the samples to the program, one of the runs was used as the reference to which the precursor masses in all other samples were aligned to. Only features comprising charges of 2+ and 3+ were selected. The raw abundances of each feature were automatically normalized and logarithmized against the reference run. Samples were grouped in accordance to the comparison being performed, and an ANOVA analysis was performed. Features with an ANOVA p-value ≤ 0.05 and a ratio>1.5 in either direction was only considered. A peak list containing the information of these significantly different features was generated and exported to the Mascot search engine (Matrix Science Ltd.).

The generated mgf file was searched against Uniprot/Swissprot human database, considering Cysteine Carbamidomethylation as fixed modification and oxidation of methionine as variable modification. 10 ppm of peptide mass tolerance and 0.5 Da fragment mass tolerance were used, and 2 missed cleavages were allowed. Spectra were searched against Uniprot/Swissprot database restricted to human entries, and only hits with a FDR<1% were kept. The list of identified peptides was imported back to Progenesis LC-MS, and the previously quantified features were matched to the corresponding peptides. Non-conflicting peptides (peptides occurring in only one protein) were specifically chosen for quantitative purposes, and only proteins with at least two quantified non-conflicting peptides were selected. The significance of expression changes was again tested at protein level, and proteins not satisfying the ANOVA p-value ≤ 0.05 and Ratio>1.5 in either direction criteria were filtered out.

### Functional analysis

GO enrichment analysis was carried out using the DAVID online tool (http://david.abcc.ncifcrf.gov/summary.jsp).[30, 31] DAVID is a GO Term annotation and enrichment analysis tool used to highlight the most relevant GO terms associated with a given gene list. A Fisher Exact test is used in order to determine whether the proportion of genes considered into certain GO term or categories differ significantly between the dataset and the background. A FDR-corrected version of the Fisher’s test p-value can be obtained and used for more conservative result selection. Biological Process (BP), Molecular Function (MF) and Cellular Component (CC) categories were assessed, and only GO Terms enriched with a p value < 0.05 were considered for comparison and discussion.

Ingenuity Pathway Analysis (IPA, QIAGEN Redwood City, www.qiagen.com/ingenuity) was used for the functional analysis of the proteins identified. The calculated p-values determine the probability that the association between proteins in the dataset and a given canonical pathway, functional network or upstream regulator is explained by chance alone, based on a Fisher’s exact test (p-value < 0.05 considered as significant). Activation z-score represents the bias in gene regulation that predicts whether the upstream regulator exists in an activated (positive values) or inactivated (negative values) state, based on the knowledge of the relation between the effectors and their target molecules. All relevant data are within the paper and its supporting information files.

## Results

### Differential analysis

**Fig 2:**
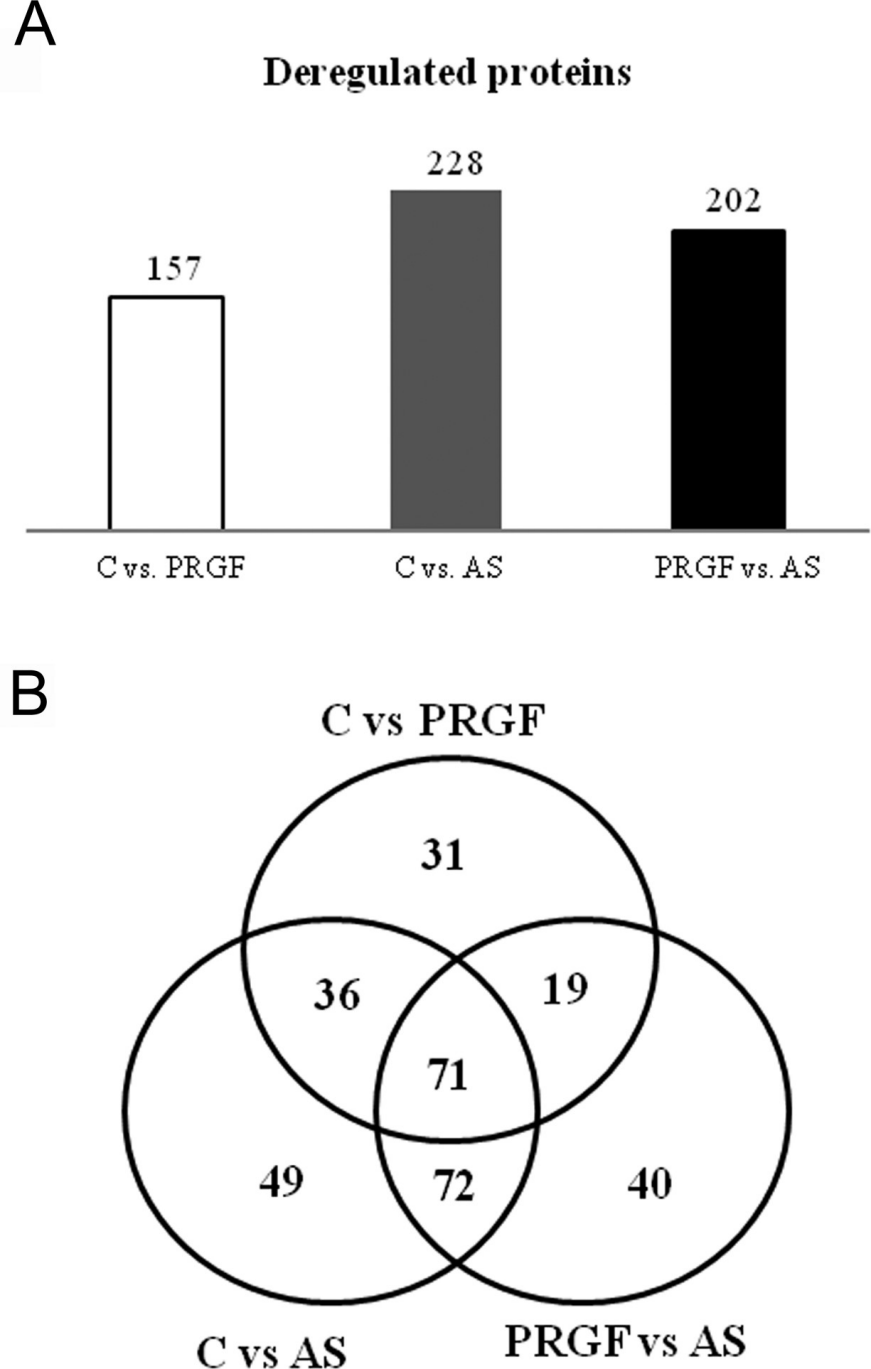
Deregulated proteins upon PRGF eye drops and AS treatment. A) Total number of differential proteins in the different comparisons performed. B) Venn diagram depicting the overlap degree between the differential proteins in each comparison.

Protein samples coming from the three conditions (PRGF, AS and control) were analyzed for differential expression using Progenesis QI software (Fig 2). Three comparisons were performed, namely Control vs. PRGF, Control vs. AS and PRGF vs. AS. Proteins with at least two non-conflictive peptides and a p value<0.05 and a ratio>1.5 in either direction and in any of the comparisons were selected for further analysis (Tables A through D of S1 File). The number of deregulated proteins in each comparison was 157, 228 and 202, respectively (Fig 2A). The Venn diagram in Fig 2B summarizes the intersection of the differential proteins from the three analyzed comparisons. After careful evaluation of the PRGF vs. AS comparison, we realized that from the 202 deregulated proteins, a total of 71 were shared by all comparisons, that is, C vs PRGF, C vs AS and PRGF vs AS. Furthermore, 72 proteins were shared by just 2 comparisons (C vs AS and PRGF vs AS) while 19 proteins were shared by the comparisons C vs PRGF and PRGF vs AS (Fig 2B).

It might be relevant to describe some of the deregulated proteins that are observed in the PRGF vs. AS comparison. For example, among the 71 shared proteins by all combinations, it is remarkable to distinguish some proteins up-regulated after AS-treatment: vinculin (VINC), integrin β1 (ITGB1) and actinin 1 (ACTN1), and other proteins as profilin 1 (PFN1) downregulated after AS-treament. Some other deregulated proteins that are shared by two of the comparisons include vimentin (VIM) (shared by C vs PRGF and PRGF vs AS), and cortactin (CTTN), actin-related protein-3 (ACTR3) and myosins like myosin-10 (MYH10) and myosin-12 (MYL12A) (shared by C vs AS and PRGF vs AS), all of them up-regulated after AS treatment. In addition, some proteins are only specific to the comparison of PRGF vs AS, as actin-related protein-2 (ACTR2) that is up-regulated in AS-treatment or septin-9 (SEPT9) that is up-regulated in PRGF-treatment (Tables A through D of S1 File).

### Functional analysis

A Gene Ontology (GO) analysis was carried out for the deregulated proteins, with the aim of characterizing roughly the functional processes these proteins are involved in. Processes enriched with a Fisher’s exact test p value<0.05 were selected for each of the protein lists and further compared (Tables E through I of S2 File). Interestingly, some GO Terms related to cytoskeleton function, such as “cytoskeleton” (p value 4.48E-06) and “regulation of cytoskeleton organization” (p value 3.98E-05) were exclusively enriched in the C vs AS dataset, being absent from the PRGF treatment. In addition, other cytoskeleton-related terms, such as “cytoskeletal protein binding” or “actin cytoskeleton” were related to both PRGF and AS treatments, but the significance of the correlation of those processes was much higher with the AS protein dataset (Tables E through I of S2 File).

**Fig 3:**
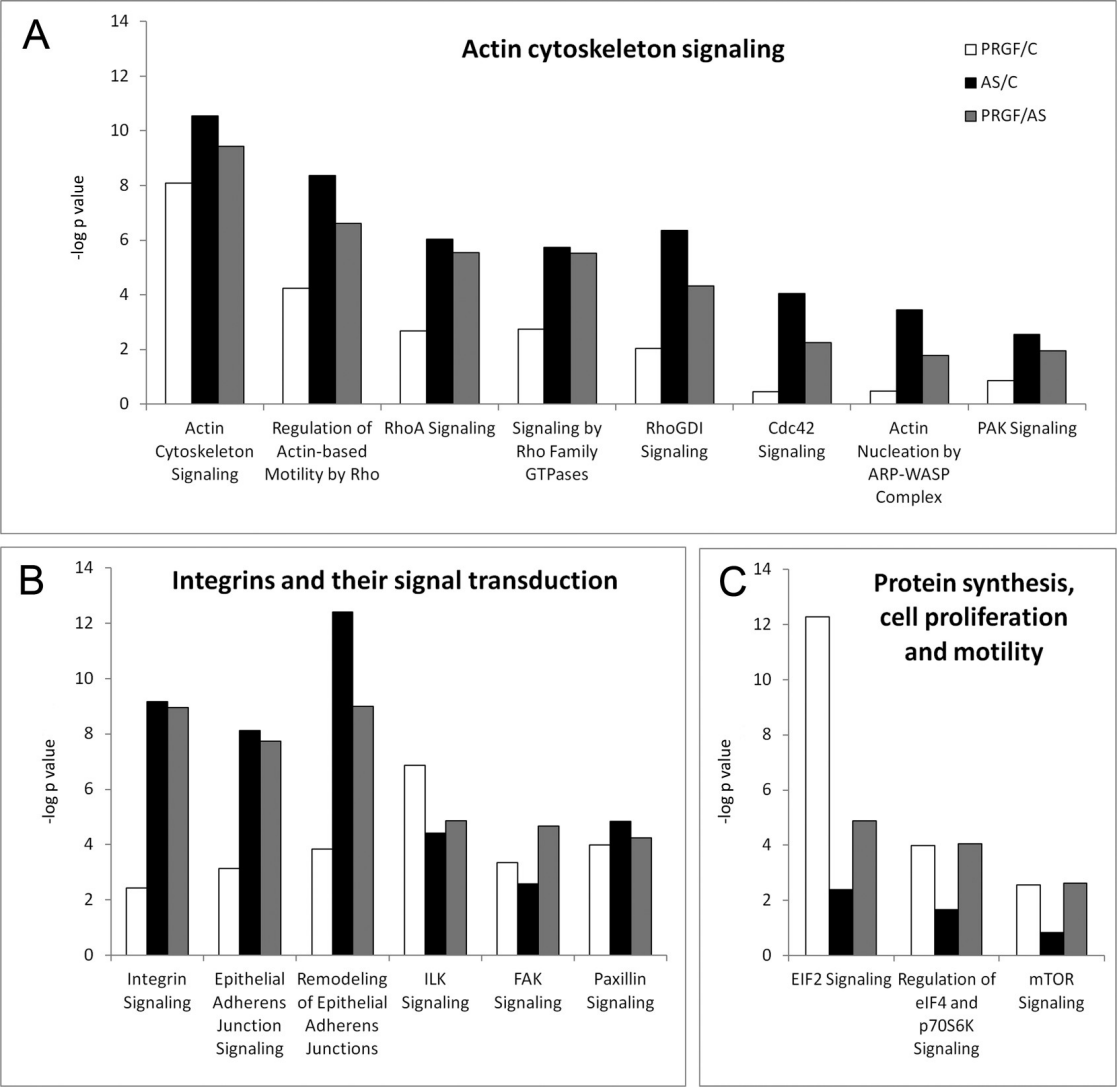
Canonical pathways analysis of the differential proteins. The most significantly enriched canonical pathways (–log p values in the y axis) are displayed. The results are clustered in functionally-related groups of processes: actin cytoskeleton signalling (A), integrins and their signal transduction (B), and protein synthesis, cell proliferation and motility (C).

IPA was accomplished for further characterizing the functional processes in which the deregulated proteins are involved (Tables J through L of S3 File). Fig 3 summarizes the canonical pathways that are most differentially associated to each of the treatments, that is, the pathways with the highest p-value difference between PRGF and AS treatments. This analysis revealed that these canonical pathways are distributed mainly in three relevant biological functions: A) Actin cytoskeleton signaling, B) Integrins and their signal transduction, and C) Protein synthesis, cell proliferation and motility. Eight of 17 canonical pathways are related to actin cytoskeleton signaling, including Actin Cytoskeleton Signaling, Regulation of Actin-based Motility by Rho, RhoA Signaling, Signaling by Rho Family GTPases, RhoGDI Signaling, Cdc42 Signaling, Actin Nucleation by ARP-WASP Complex, and PAK Signaling, (Fig 3A). Again, the significance of the association between the proteins deregulated upon AS treatment and these cytoskeleton-related terms is higher than with PRGF, suggesting a tight correlation between AS and cytoskeletal functions. Six of the canonical pathways (Integrin Signaling, Epithelial Adherens Junction Signaling, Remodeling of Epithelial Adherens Junctions, ILK Signaling, FAK Signaling and Paxillin Signaling) are related to integrins and their signal transduction (Fig 3B). Integrin signaling seems to be more strongly correlated to AS treatment, although ILK signaling and FAK signaling are more significant among the PRGF-deregulated proteins. Finally, the rest 3 of 17 pathways (EIF2 Signaling, Regulation of eIF4 and p70S6K Signaling and mTOR Signaling), are associated with protein synthesis, cell proliferation and motility (Fig 3C), and are more significantly related to PRGF-mediated response.

**Fig 4:**
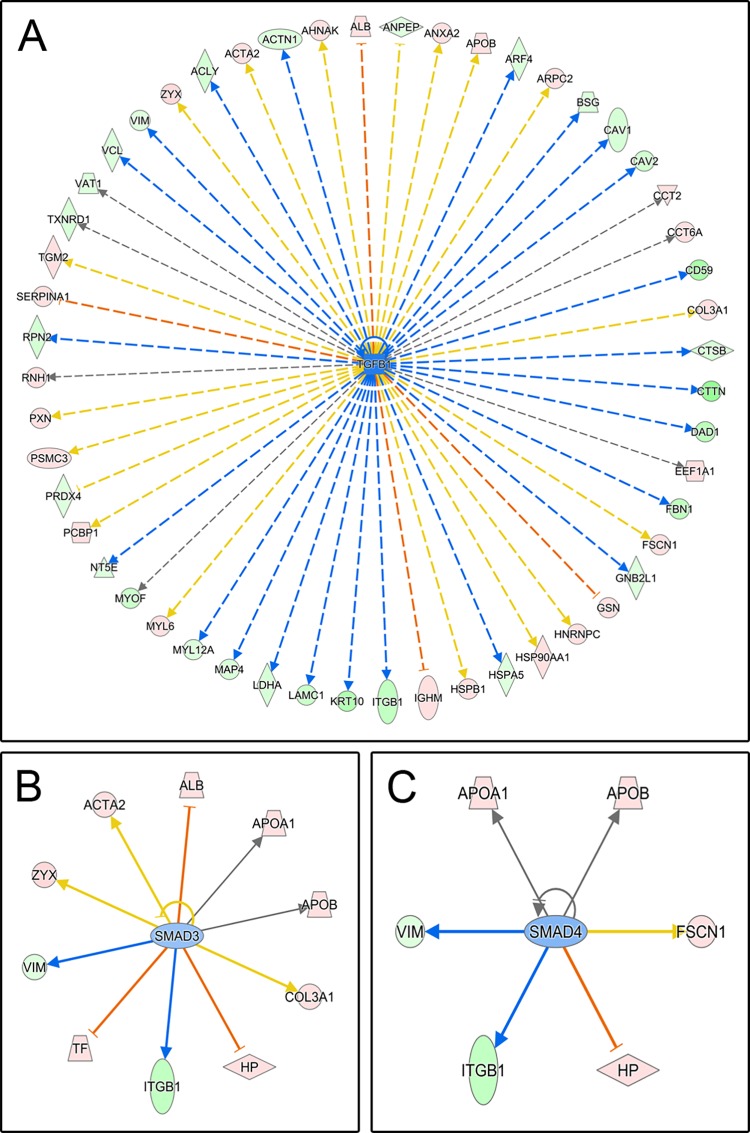
Upstream regulators analysis. IPA displays the likely molecular effectors of the protein deregulation observed. TGF-β1 (A), SMAD3 (B) and SMAD4 (C)-related proteins are displayed.

Furthermore, the IPA upstream regulators analysis revealed a differential activation of the TGF-β1 pathway depending on the treatment. The expression pattern of the deregulated proteins upon PRGF or AS treatment revealed a strong correlation with TGF-β1 signaling (-log p value 10.69 and 19.25, respectively), suggesting an important signaling mediated by this cytokine for the phenotype of these cells. But in addition to this observation, the direct comparison between PRGF and AS-treated cells revealed that the degree of activation was different, suggesting that TGF-β1 is inhibited (or less activated) in PRGF-treated cells (Fig 4A, z-score -1.51). The activation state of additional TGF-β1-related upstream regulators, such as SMAD-3 (Fig 4B, z-score -0.55) and SMAD-4 (Fig 4C, z-score -0.65) is in agreement with the mentioned inactivation process of TGF-β1 after PRGF treatment in comparison to AS.

## Discussion

In the present study the protein expression patterns induced by PRGF and AS eye drops treatment over human myofibroblasts have been characterized. Human keratocytes were treated with TGF-β1 in order to induce their differentiation towards myofibroblasts. Then, these cells were treated with either AS or PRGF and protein expression patterns were analyzed by means of mass-spectrometry.

According with previous observations, our results provide molecular evidences consistent with a myofibroblastic differentiated phenotype in AS treated-cells, whereas PRGF-treated cells show attenuation on this phenotype. Previous results from our group demonstrated that PRGF inhibits and reverts TGF-β1-induced myodifferentiation by diminishing the levels of the myofibroblastic phenotype characteristic proteins α-actin (α-SMA), vimentin and desmin in conjunctival fibroblasts and keratocytes regarding to autologous serum [32–34]. Consistent with this idea, many down-regulated proteins upon PRGF treatment, such as vimentin, vinculin, cortactin and actin related proteins -2 and -3 (ARP2 and ARP3 respectively), are cytoskeletal proteins present in the myofibroblastic phenotype. Cortactin and α-actinin are involved with actin-assembly and remodeling [35] and ARP2 and ARP3 are involved in the organization of the actin cytoskeleton and serve as nucleation sites for new branched actin filaments [36]. Vinculin is a cytoskeletal protein associated with cell-cell and cell-matrix junctions and vimentin is a major constituent of the intermediate filament family of proteins that acts during corneal remodeling [34]. Another interesting protein, profilin 1, is an actin-binding protein that at low concentrations enhances polymerization of actin; in this case, this protein is down regulated in AS treated cells, suggesting that AS could favor an elevation in actin levels.

The functional analyses carried out also point in this direction. Proteins that are downregulated upon PRGF treatment or that are more abundant in AS-treated cells were clustered into three main canonical pathway groups in the IPA analysis: A) actin cytoskeleton signaling (8 canonical pathways), B) integrins and their signal transduction (6 canonical pathways), and C) protein synthesis, cell proliferation and motility (3 canonical pathways). This suggests a close relationship between AS-treated cells and cytoskeletal functions (Fig 4A and 4B). On the other hand, proteins upregulated upon PRGF-treatment or that are less abundant in AS-treated cells reveal a greater association with processes such as protein synthesis, proliferation and cellular motility (Fig 4C), mainly related to EIF2 signaling (eukaryotic translation initiation factor 2 alpha), EIF4 regulation (eukaryotic translation initiation factor 4) and mTOR signaling (mammalian Target of Rapamycin). Therefore, myofibroblastic phenotype protein profiling is reduced in PRGF-treated cells. Several studies have demonstrated that an alteration in EIF2 pathway is related to the development of neurodegenerative, metabolic and cancer diseases [37, 38], while EIF4 is a protein complex involved in mRNA translation implicated in cell proliferation [39]. In addition, mTOR is involved in the control of mRNA transcription, ribosome formation, cell cytoskeleton organization, cell membrane transport, regulation of cell growth, proliferation and death [40–42]. Our results provide a link between PRGF-Endoret function and protein synthesis, cell proliferation and motility, since these results are more significantly related to this condition.

Inhibition of TGF-β1 activity might be the mechanism for the phenotype reversion upon PRGF treatment. In vitro studies from our group showed that PRGF-Endoret inhibits and reverts TGF-β1 induced myodiferentiation in gingival fibroblasts as well as in keratocytes and conjunctival fibroblasts [32, 43]. There is also evidence that PRGF-Endoret treatment reduces the number of myofibroblasts in a corneal wound healing animal model [20]. TGF-β1 is involved in hereditary corneal dystrophies such as type II granular corneal dystrophy (GCDII) [44], and it is related to glaucoma and inflammatory processes [45]. Furthermore, there is as well a correlation between TGF-β1 tear levels and corneal haze formation in corneal refractive surgery [46]. Therefore, the modulation of TGF-β1 expression through the use of PRGF eye drops arises as a plausible strategy to treat several ocular surface pathologies.

The expression patterns analyzed in this work are consistent with this idea. PRGF or AS-treated cells revealed a solid relationship with TGF-β1 signaling, which is necessary for the stromal keratocytes due to its implication in the conversion of fibroblasts to myofibroblasts, but this association is much more consistent in AS-treated cells, suggesting that TGF-β1 might be less active in PRGF-treated cells. Our results come along with previous scientific evidence that supports that PRGF participates in myofibroblastic reversion to fibroblasts, achieving corneal transparency in corneal lesions [21].

Although this preliminary study has certain limitations due to the low number of donors, the proteomic analysis performed could help to understand the molecular events underlying AS and PRGF-driven tissue regeneration processes, providing novel data that agrees with the modulation of TGF-β1 activity and the reversion of the myofibroblastic phenotype by PRGF described before.

## Supporting information

S1 File Tables A through D. Differential protein expression data.
(XLSX)

S2 File Tables E through I. Gene Ontology analysis.
(XLSX)

S3 File Tables J through L. Summary of IPA analysis results.
(XLSX)
